# Supplementary material for: Teucrium polium (L.): Phytochemical Screening and Biological Activities at Different Phenological Stages
Source: Molecules. 2022 Feb 25;27(5):1561. doi: 10.3390/molecules27051561 (PMC8911654; doi:10.3390/molecules27051561)

Teucrium polium (L.): Phytochemical Screening and Biological Activities at Different Phenological Stages

Majid Sharifi-Rad[1*], Pawel Pohl[2*], Francesco Epifano[3], Gokhan Zengin[4], Nidal Jaradat[5] and Mohammed Messaoudi[6,7]

[1] Department of Range and Watershed Management, Faculty of Water and Soil, University of Zabol, Zabol 98613-35856, Iran; Majidsharifirad@uoz.ac.ir (M.S-R.)

[2] Department of Analytical Chemistry and Chemical Metallurgy, Faculty of Chemistry, University of Science and Technology, Wyspianskiego 27, 50-370 Wroclaw, Poland; pawel.pohl@pwr.edu.pl (P.P.)

[3] Dipartimento di Farmacia, Università "Gabriele d'Annunzio" Chieti-Pescara, Via dei Vestini 31, 66100 Chieti Scalo (CH), Italy; fepifano@unich.it (F.E.)

[4] Physiology and Biochemistry Research Laboratory, Department of Biology, Science Faculty, Selcuk University, Konya 42130, Turkey; gokhanzengin@selcuk.edu.tr (G.Z.)

[5] Department of Pharmacy, Faculty of Medicine and Health Sciences, An-Najah National University, Nablus, P.O. Box. 7, Palestine; nidaljaradat@najah.edu (N.J.)

[6] Nuclear Research Centre of Birine, P.O. Box 180, Ain Oussera, Djelfa 17200, Algeria; messaoudi2006@yahoo.fr (M.M.)

[7] Chemistry Department, University of Hamma Lakhdar El-Oued, B.P.789, El-Oued 39000, Algeria.

* Correspondence: Majidsharifirad@uoz.ac.ir (M.S-R.); Tel.: +98-5432240568 (M.S-R.); pawel.pohl@pwr.edu.pl (P.P.)

Figure S1. The exemplary GC MS chromatogram of the prepared extracts (the labels given for the chromatographic peaks correspond to those given in Table 1).

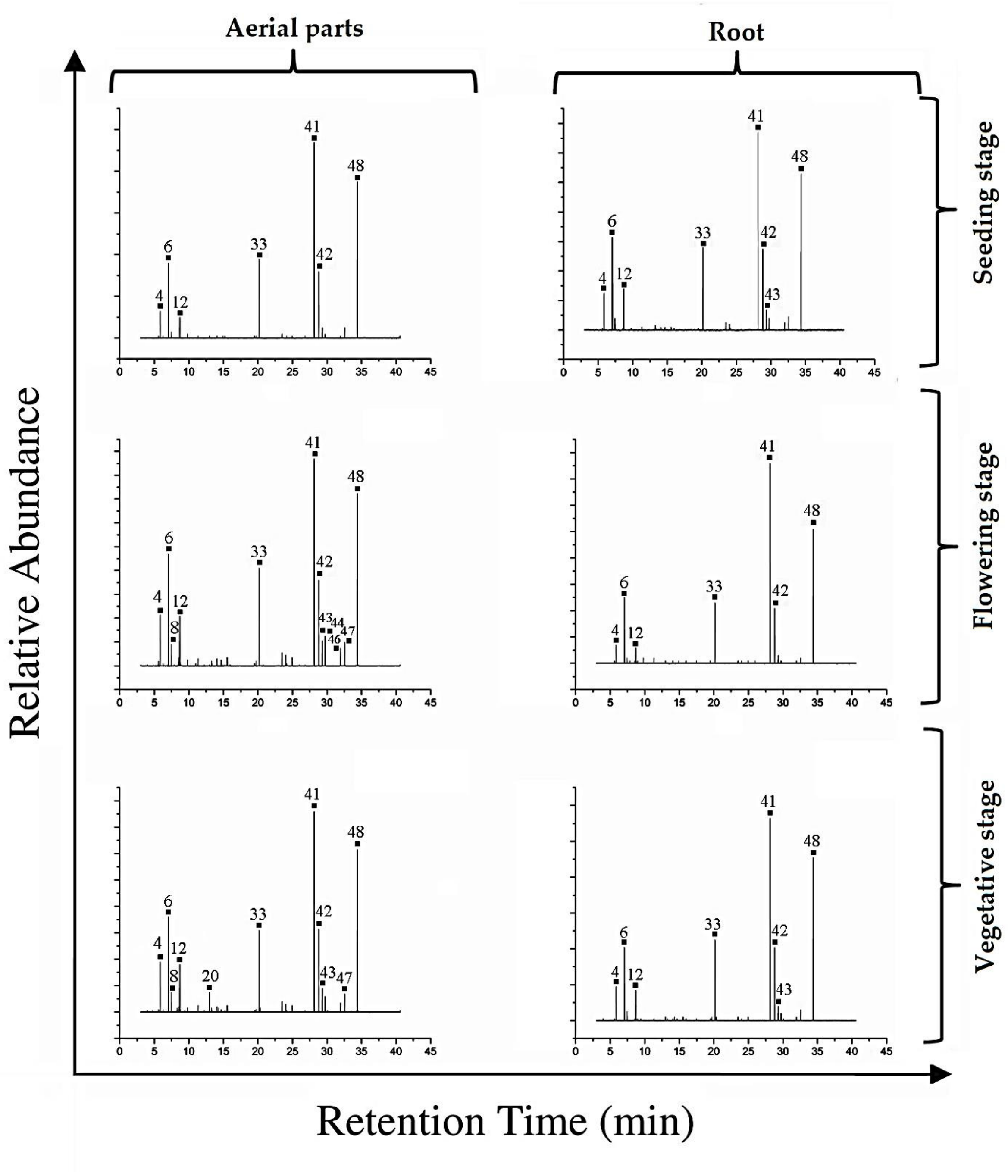

Supplement: Supplementary file 1 [file molecules-27-01561-s001.zip › molecules-1583836-supplementary.pdf]
